# Acceptability and feasibility of integrating psychosocial stimulation interventions in the inpatient care of children with severe acute malnutrition in resource-poor settings: a qualitative study

**DOI:** 10.1017/jns.2024.27

**Published:** 2024-08-02

**Authors:** Tesfalem Teshome Tessema, Andamlak Gizaw Alamdo, Eyoel B. Mekonnen, Tewodros G. Yirtaw, Fanna A. Debele, Teklu Gemechu, Tefera Belachew

**Affiliations:** 1 School of Public Health, St. Paul’s Hospital Millennium Medical College, Addis Ababa, Ethiopia; 2 SANKOFA Research and Consulting Plc, Addis Ababa, Ethiopia; 3 Jimma University College of Education, Jimma, Ethiopia; 4 Jimma University College of Public Health and Medical Sciences, Nutrition and Dietetics, Jimma, Ethiopia

**Keywords:** Psychosocial stimulation, Malnourished children, Acceptability, Feasibility

## Abstract

Children with Severe Acute Malnutrition (SAM) are at risk of developmental problems. Psychosocial stimulation can improve the developmental outcomes of hospitalised children with SAM. However, the intervention has remained underutilised in health facilities in resource-poor settings. Moreover, there is limited evidence on the acceptability and feasibility of the intervention. We conducted a qualitative study to explore the acceptability and feasibility of integrating psychosocial stimulation interventions in the inpatient care of children with SAM in selected areas in the Silti Zone, Central Ethiopia. Nine focus group discussions and 15 key informant interviews were conducted with parents, health workers, and other stakeholders. The data were transcribed, translated, and analysed using a thematic approach. Caregivers and health workers had positive attitudes toward the intervention and perceived it beneficial for the children’s development, recovery, and bonding with the mothers. Health workers reported barriers such as lack of materials, time, and space, capacity building training, and supervision for the effective implementation of the intervention. At the household level, gendered factors such as the housework burden of mothers and the inadequate engagement of fathers in childcare were the main challenges to the implementation of the intervention. Overall, psychosocial stimulation intervention was found to be acceptable and feasible for routine implementation with inpatient care provided for children with SAM. The study recommends supporting health facilities, health workers, and partners with the necessary resources and skills to promote the implementation of stimulation interventions along with the existing care provided in health facilities in resource-poor settings.

## Introduction

Severe acute malnutrition (SAM) is a global life-threatening condition that affects millions of under-five children^(1)^. Ethiopia is one of the countries with the highest burden of SAM in the world, with an estimated 400,000 children suffering from SAM in 2020^(2)^. Based on the recommendation of the World Health Organization (WHO) and the Ethiopian Federal Ministry of Health (FMoH), the management of SAM involves both inpatient and outpatient care, depending on the severity and the associated complications of the condition. A SAM child with medical complications or failed appetite tests or referred from the Outpatient Therapeutic Program should be admitted to a dedicated nutrition unit and given medico-nutritional care and support^(3,4)^. The integration of psychosocial stimulation interventions into the inpatient management of SAM children is also recommended to reduce the risk of intellectual disability and emotional impairment and promote development and growth in the rehabilitation phase^(3–5)^.

Psychosocial stimulation intervention refers to the provision of responsive care, play, communication, and learning opportunities that enhance children’s cognitive, social, and emotional development^(6)^. The intervention is particularly important for children hospitalised for severe malnutrition in early childhood. According to some scholars, these children were found to have long-term deficits in cognitive development and school achievement up to adolescence^(7)^, owing to the effect of undernutrition on the structural and functional impairment of the brain and central nervous system^(5)^.

Studies conducted in Jamaica, Bangladesh, and Ethiopia confirmed the positive contribution of psychosocial stimulation interventions in improving the development outcome of children hospitalised with SAM^(8–10)^. However, psychosocial stimulation intervention has not been adequately implemented as part of the inpatient management of children with SAM, particularly in health facilities in resource-poor settings including in Ethiopia. A study conducted in South Africa reported that play and stimulation were not implemented in the inpatient management of severe malnutrition in under-resourced rural hospitals^(11)^. Moreover, evidence on the perceptions of parents and health workers towards the stimulation intervention and the feasibility of implementing the intervention is limited globally. A qualitative study conducted in Malawi to understand the perceptions and experiences of caregivers who participated in the Kusamala Program, a 4-day hospital-based counselling programme for caregivers of children with SAM that integrates nutrition, water, sanitation, hygiene, and psychosocial stimulation, reported the positive sentiments and experiences of the parents towards the programme^(12)^. A feasibility study of the Kusamala Program identified caregivers’ perceived value as a potential barrier to the implementation of the programme. Prioritisation of other ward activities and shortages of staff were also identified as constraints for the health workers^(13)^. In the studies that tested the stimulation intervention among hospitalised SAM children, the WHO recommendations on stimulation interventions were not followed^(8–10,12)^. Therefore, the implementation practice and the feasibility of the WHO recommendations on psychosocial stimulation interventions for children hospitalised with SAM were unclear, especially in low-resource contexts^(14)^. A systematic review published in 2017 has reported feasibility concerns on the stimulation interventions used in studies conducted in Jamaica and Bangladesh^(14)^.

Therefore, we have been conducting a Cluster Randomized Control Trial to examine the effects of psychosocial stimulation on the development, nutrition, and treatment outcomes of hospitalised SAM children In Ethiopia (EPSoSAMC Study), following the recommendations of WHO and FMoH^(3,4)^. This qualitative study was included in the trial to explore the acceptability of the stimulation intervention among parents and health workers who participated in the implementation of the intervention. Another aim of the qualitative study was to understand the feasibility of implementing the WHO recommendations on psychosocial stimulation interventions for children hospitalised with SAM in resource-poor health facilities. Our findings could generate relevant information on how best the intervention would be integrated, the financial, material and human resources required, the training needs, and how best to ensure the follow up of children and their families^(15)^. Moreover, it could enable the identification of the barriers and facilitators that affect the routine implementation of the stimulation intervention in the care and support of hospitalised SAM children.

## Methods

### Study area

Based on the new administrative establishment, the Silti Zone is one of the administrative divisions of the Central Ethiopia Regional State. It has a population of 1,020,337^(16)^ and agriculture is the main source of livelihood for about nine out of ten people in the Silti Zone^(17)^. Malnutrition is one of the major causes of childhood deaths in Ethiopia. The prevalence of stunting, wasting, and underweight in the country was 37%, 7%, and 21%, respectively^(18)^. The present study was carried out among health facilities located in Hulbareg Woreda, Silti Woreda, and Worabe Town Administration, which represent various socio-demographic disparities in the Zone.

### Psychosocial stimulation interventions

The study is part of a Parallel-Group Cluster-Randomized Controlled Trial (EPSoSAMC Study) that has been underway in the Siliti Zone, Central Ethiopia. The trial aimed to examine the effect of psychosocial stimulation interventions on the development, growth, and treatment outcome of hospitalised SAM children aged 6–59 months [Pan African Clinical Trials Registry: PACTR201901730324304]. The trial involved health facilities (clusters) that provide inpatient care for children with SAM. The health facilities were randomly assigned into intervention or control groups, and trained health workers were responsible for the recruitment of eligible SAM children admitted to the facilities. In the intervention facilities, dedicated play corners were established and equipped with age and developmentally appropriate play materials. Therefore, children were given psychosocial stimulation interventions following the recommendations of the WHO and FMoH. In these facilities, trained intervention workers (Play guides) recruited from the local areas facilitate a half-hour stimulation session daily after the initial phase of treatment for children. In the six-month follow up period, intervention workers visit children five times (at the end of the 1st week, 2nd week, 1st month, 3rd month, and 6th month) based on the WHO’s recommended follow up schedules. In each visit, intervention workers engage children in a half-hour stimulation session. In the control health facilities, children were given routine inpatient care and home visits without psychosocial stimulation. All primary caregivers of SAM children were also given counselling on health, nutrition, development, and related topics during the inpatient and follow up periods. Further details of the trail have been presented in previous publications^(19)^.

### Participants and data collection

We conducted a qualitative study using Key Informant Interviews (KIIs) and Focus Group Discussions (FGDs) between November and December 2022. Nine FGDs that lasted 1:15—1: 45 hours were conducted among a homogenous group of 6-9 mothers and fathers of children who received psychosocial stimulation intervention as part of their inpatient care. Fifteen Key Informant Interviews (KIIs) lasting 30-45 minutes were also conducted with health workers and intervention workers that deliver the stimulation intervention, Health Extension Workers (HEWs), Woreda and Zonal Health Office staff, and the staff of Worabe University. The saturation of information was used to judge the adequacy of the KIIs and FGDs. A purposive sampling technique was employed to select KII respondents and a convenience-sampling technique was used for the selection of FGD participants. Semi-structured KII and FGD Guides were used to guide the data collection where probing questions were included in the tools to allow getting detailed information. Data on the perception of the parents about the benefit of the stimulation intervention, their adherence to the recommended stimulation practices, and the barriers and facilitators for implementing the interventions including family support were collected. Moreover, the experience of those directly and indirectly involved in the implementation of the intervention, and the potential challenges and opportunities in the implementation of the stimulation interventions with the routine care given in the nutrition units were also explored. The data were collected in the local language (Amharic). All interviews and discussions were tape-recorded with the consent of participants and notes were also captured during the data collection. To ensure the research participants’ privacy, data were collected in private areas such as in the compounds of health facilities and offices and no one else was present in the interviews and discussions except the study subjects and the data collection personnel. Two public health researchers (TT and AA) who are members of the main trial team collected the data. TT was responsible for facilitating the interviews and discussions, while AA was in charge of taking notes and recording. They have MSc. level training in public health with additional training in qualitative research methods. They also have demonstrated experiences in qualitative data collection and knowledge of local context. They maintained neutrality and consistency throughout the data collection process to enhance the validity and reliability of the data. Although they have an interest in the subject matter, their background does not influence the collected data. The involvement of other authors in different stages of the study could have also minimised the potential introduction of biases owing to the background of the researchers engaged in the data collection. Moreover, the finding of the main trail was not known during the collection and analysis of the data. The whole process of the study followed the recommendations included in the Consolidated Criteria for Reporting Qualitative Research^(20)^.

### Data management and analysis

Before the actual analysis, the data collected in a digital recorder were transcribed into written form and translated into English. The thematic approach was used to analyse the data following the six steps namely: familiarisation, coding, generating themes, reviewing themes, defining and naming themes, and writing up. Themes or patterns within the data were identified using a combination of inductive and deductive approaches^(21)^. Two researchers (TT and AA) independently read the transcripts and assigned initial codes. Once all codes were identified, they were then discussed, and similar codes were collated together. The codes were then sorted and collated into potential themes, which were reviewed in relation to the research objective to ensure the identified themes represented the data. Finally, the themes were defined and named as presented in the below section. In the analysis process, no specific software was used. The five main themes derived from the data include 1) The perception of parents about SAM and its impact on child health, growth, and development: 2) The concerns of parents about the future of their children: 3) The perception of psychosocial stimulation intervention and its benefits: 4) The acceptability of psychosocial stimulation interventions: and 5) The feasibility of incorporating psychosocial stimulation interventions into the inpatient care of children with SAM.

## Result

A total of 63 parents (Mothers-43 and Fathers- 21) participated in the FGDs. Fifteen interviews were also facilitated with health workers involved in psychosocial stimulation interventions (6), HEWs (2), intervention workers (2), Woreda Health Office staff (2), Zonal Health Office staff (2), and the staff of Worabe University (1). In the section below, the key findings of the study are presented based on the five identified themes, and illustrative quotes stated by the participants are presented to elaborate on issues in more detail.

### The perception of parents on SAM and its impact on child health, growth, and development

Parents were asked several questions to explore their understanding of SAM. Issues such as the causes of SAM, the consequences of SAM on the health, growth, and development of children, and their understanding of the required treatment were explored. Some parents showed an adequate understanding of SAM and mentioned details about its effects on the health, growth, and development of children. They voiced inappropriate feeding practices as the major cause of the problem and cited special milk and medicine as the key elements for treating their children in the health facilities. However, there were still parents who had a limited understanding of SAM and how to prevent it.

It is a condition that affects the health of children making them very thin or swollen. It is caused by a lack of food. These children are treated with special food and medicine in the health facilities. Feeding children in a better way prevents SAM from happening (FGD, Mother).

Nutrition and health education were provided to all parents whose children were admitted for inpatient care in the stabilisation centre (SC). Therefore, we attempted to explore the parents’ understanding of SAM before their children were admitted to the SC to understand the improvement in their knowledge that could be attributed to the education interventions implemented in the health facilities. Accordingly, nearly all parents, including those with an adequate level of understanding at the time of data collection, universally acknowledged that they did not know the details of SAM until their children were admitted to the health facility.

I did not know what SAM was until health workers told me in this hospital. They measured my child and said he has SAM. I thought my child was just thin.” (FGD, Mother)

Health workers have also expressed similar sentiments, mentioning that most parents lack a basic understanding of the problem when their children are first diagnosed with SAM. Therefore, it could be sound to consider the valuable contribution of health education intervention in terms of improving the understanding of SAM among parents who participated in the study.

This study also attempted to explore community-based beliefs and practices related to SAM in children. The participants shared various beliefs and practices related to SAM that could have influenced community wide behaviours and decisions, including health-seeking practices for children affected by SAM. Attributing SAM to supernatural causes, such as evil spirits, curses, or God’s /Allah’s will was among the issues identified in the community. Traditional and spiritual remedies from traditional healers and spiritual people were also mentioned among practices often considered before or instead of seeking medical care for children with SAM.

My child suffers from SAM, which I believe was caused by a curse. He used to be healthy, but then he began to lose weight and fall ill frequently. I took him to a traditional healer who prescribed him some herbs [FGD, Mother]

In addition to the community-based beliefs and practices, some participants also expressed dissatisfaction with the health system and the health workers when explaining why the community prefers going to the traditional and/or spiritual people rather than seeking help from the available health facilities.

We are not getting good service in the health facilities. They just give them some medicine and food and send us home. They do not explain anything to us except to tell us the child has experienced a food shortage [FGD, Mother].

### The concerns of parents about the future of children with SAM

The study revealed that most parents have various concerns about the future of their children with SAM. They expressed concerns about their children’s survival, recovery, growth, and health. The fact that their children had difficulties in speaking, playing, and socialising compared with other children of their age was mentioned to have exacerbated their concerns about their children’s future. Moreover, some participants mentioned their concern that their children would face stigma and discrimination owing to their physical conditions in their community.

I worry that my child will be behind the development of other children. He is more than one year old but he has difficulty walking properly [FGD, Father].

### The perception of psychosocial stimulation intervention and its benefits

This theme explores how parents whose children received psychosocial stimulation interventions as part of inpatient care and health workers perceived psychosocial stimulation interventions and the benefits of the intervention. Accordingly, most parents showed a good understanding of the interventions. They described psychosocial stimulation as the provision of care, love, and attention to children through activities such as talking, singing, and playing with children. They further expressed positive attitudes toward psychosocial stimulation interventions and recognised the importance of the intervention in terms of improving the condition of their children, enhancing the interaction of the children with their mothers, and increasing the knowledge and skills of the parents. Improvement in the appetite, weight gain, and health status of children, their skill and activity level, and the reduction of stress among parents and children were further mentioned among the benefits of the intervention.

Psychosocial stimulation is giving love and care to children. It is talking to them, playing with them, and making them feel happy and safe. It helps me to bond with my child and better understand him to meet his needs [FGD, Mother].

I see differences in my child after stimulation interventions. He is now interacting more with other children and staff [FGD, Mother].

A health worker added

The psychosocial stimulation intervention is very beneficial for the relationship between children and caregivers. It encourages them to play together, talk to each other, and share their feelings. It also helps caregivers to better understand their children and show love and care [KII, Health Worker, Male].

The observed good level of understanding of the intervention and the benefit among most parents could be attributed to the educational activities that were integrated into the psychosocial stimulation interventions. Health workers have also stated that the intervention has improved the understanding of parents about the problem of SAM children and their treatment process because of their engagement in the delivery of the intervention. They further reported the benefit of the interventions in terms of enhancing treatment outcomes for children. According to the respondents, such interventions would have a long-lasting benefit of improving the care given to children, thereby preventing the re-occurrence of SAM.

Psychosocial stimulation intervention helps children to recover faster. It helps them to eat more, gain weight, and get healthier [KII, Health Worker, Male].

### The acceptability of psychosocial stimulation interventions

This study attempted to explore the experience of parents who were engaged in psychosocial stimulation intervention of their SAM children as part of the medico-nutritional care provided in the SC. Similarly, health workers were also consulted to share their experience of delivering psychosocial stimulation interventions for children with SAM. It was revealed that parents and health workers shared both positive and negative experiences in the implementation of the interventions.

Most parents expressed positive experiences with participating in psychosocial stimulation interventions for children with SAM, although few of them also shared unpleasant experiences. Because play was the major intervention introduced into the routine medico-nutritional care of SAM children, parents described the intervention as fun, interesting, and rewarding activities that they looked forward to doing with their children. The positive progress, such as positive changes in the children’s behaviour, mood, and performance the parents have seen in their children and their enhanced interactions with their children were mentioned to have contributed to the positive experience reported by parents

I enjoy doing psychosocial stimulation with my child. It is fun and interesting. We play games and sing songs. We laugh and have a good time together [FGD, Mother].

However, similar to most of the parents, nearly all of the health workers and intervention workers who facilitated the interventions also reported their positive experiences in delivering the intervention. One of the health workers mentioned the following underlining the motivation brought by the intervention and the associated better outcome seen in SAM children among his team members.

… The intervention is, I think, rewarding. Compared to the time before, I have seen changes in SAM children. I see them improving their skills and becoming more active and engaged, which seems encouraging [KII, Health worker, Female]

The parents and health workers perceived the intervention to be relevant in terms of benefiting children with SAM. This possibly indicates the acceptability of the psychosocial stimulation interventions among the parents of children with SAM and health workers who have a crucial role in caring, feeding, stimulating, and supporting children during and after hospitalisation.

### The feasibility of incorporating stimulation interventions into inpatient care of children with SAM

In this section, how psychosocial stimulation interventions could be adapted to fit the local context and the key barriers and opportunities that influence the effective implementation of the interventions are presented with due emphasis on the existing capacity of the health facilities operating in resource-limited settings. Health workers identified several factors that confer the opportunity for providing the intervention to hospitalised SAM children in the area. The alignment of stimulation interventions with the national guidelines for SAM management, the availability of functional SC at the level of health facilities with health workers and community health workers to deliver interventions at the health facility and community level, and the relative cost-effectiveness of the intervention were reported. In the same manner, challenges such as the lack of standards to guide the implementation of the intervention, material resources, human resources, time, tools, and the limited priority given to the intervention by the health system were repeatedly reported among the major challenges. Health workers mentioned that, except for information available in the few national documents, there were no documents such as guidelines or manuals to guide the standardisation and appropriate implementation of the intervention.

We do not have enough information about the psychosocial stimulation intervention. No protocol or manual is available to guide the health workers and the health facilities in setting up and implementing the intervention [KII, Health Worker, Male].

Moreover, the lack of adequate resources, including play materials, space/ play area, and other essentials needed to administer the intervention was also mentioned among the challenges.

The intervention needs resources and toys that are suitable for different ages and abilities of children. In most of the health facilities in our area, we do not have the required materials to do psychosocial stimulation with the children [KII, Woreda Heath Office Staff, Male].

We do not have enough space to facilitate psychosocial stimulation with the children. As you can see, we are using this room, which is very small [KII, Heath Worker, Female].

The shortage of health workers in most health facilities was also mentioned as a challenge for the successful implementation of the intervention. Moreover, the lack of capacity-building training on psychosocial stimulation interventions, except for the very basic elements included in the training of SAM management was also stated.

In our Woreda, we have a staff shortage. We only have a few health workers who provide nutrition services who are busy and overwhelmed making it challenging to implement the intervention regularly [KII, Woreda Heath Office Staff, Male].

… To my knowledge, no capacity-building training was provided to the health workers on the psychosocial stimulation intervention except those trained through the research project implemented in our Zone [KII, Health Worker, Male].

Time was also found to be the major limitation on behalf of the health workers. The fact that the interventions demand dedicating several hours daily to administer the intervention and the additional time needed to prepare play materials, educate mothers about how to stimulate their children, and the related issues were mentioned as challenging. A health worker who participated in the delivery of the interventions reported the following when asked to share his thoughts about difficulties in delivering psychosocial stimulation interventions for children hospitalised with SAM.

Psychosocial stimulation is a time-intensive intervention. We have too many other tasks and responsibilities to attend to daily [KII, Health Worker, Female]

The lack of adequate supervision and support to enable health workers to practice psychosocial stimulation interventions consistently and the lack of accountability mechanisms or incentives were also reported as challenges. Some health workers linked the lack of tools, capacity building initiatives, and accountability and supervision mechanisms toward psychosocial stimulation interventions with the inadequate priority given to the intervention at different levels of the health system. Lastly, the resistance or reluctance from some caregivers was also reported as a challenge in implementing psychosocial stimulation interventions.

The study has attempted to explore whether the interventions would be feasible to the parents, particularly mothers of the children, who could play a crucial role in providing a nurturing and stimulating environment for their children, and about the potential challenges that could hinder their ability to implement the interventions effectively and consistently. Among others, some social and gendered norms, such as the disproportionate household burden of women were reported to influence the intervention. Women in the area spend most of their time doing multiple household chores such as fetching water and firewood, cooking, cleaning, washing clothes, and caring for children. Hence, owing to their competing priorities, they may not have adequate time to stimulate their children at home. The lack of support from husbands and the inadequate engagement of men in the care of children were further mentioned to further limit the effective implementation of the stimulation intervention at the household level.

Here, I am doing everything based on the direction of the health workers, as I have nothing to do except care for my sick child. This is not the case at home. I have many household responsibilities. How would I spend this much time playing with my kid and preparing his play materials? [KII, Mother].

Some respondents have also mentioned community-based misconceptions such as associating SAM with spiritual beliefs such as the will of God/ Allah or a curse to influence the mothers’ intention, behaviour, and confidence in implementing the psychosocial stimulation interventions in a home setting. Lastly, health workers and those who participated from Woreda and Zonal Health Offices were asked about the potential feasibility of integrating the interventions with the routine medico-nutritional care given to hospitalised SAM children in the SC. According to most of the respondents, the intervention was viewed as feasible. However, a well-tailored mechanism to address the potential demand and supply side challenges relevant to the intervention should be designed to ensure the feasibility of the intervention in the area.

## Discussion

The study has shown that the parents of children hospitalised for SAM lack adequate understanding about the problem of their children and its medical aspects until their children are diagnosed in the health facilities. Parents expressed concerns about the future health and growth of their children mainly because of the difficulties they experienced. Parents also stated community-based beliefs that attributed SAM to supernatural causes and expressed concerns that their children would face stigma or discrimination in the community. The community-based practice of using traditional and spiritual measures to manage SAM has also been reported.

Parental knowledge and practices are crucial factors in preventing and managing SAM among children as they have a key role in providing adequate nutrition, care, and health services to their children^(22–24)^. The understanding of parents about SAM and its impact on child health, growth, and development in Ethiopia is not well documented in the literature. However, the available studies suggest that many parents lack sufficient knowledge or skills to recognise the signs and symptoms of SAM, initiate appropriate feeding practices, seek timely medical attention, or follow up with treatment and rehabilitation programmes^(25,26)^.

All parents reported that they had never heard of psychosocial stimulation interventions before the admission of their SAM children to the SC. However, most parents showed a good understanding of the interventions and expressed positive attitudes. The health education provided in integration with the interventions and the active engagement of parents in the delivery of the stimulation intervention were repeatedly mentioned by the health workers to have contributed to the observed level of understanding among parents. Health workers involved in the feasibility study of the Kusamala Program expressed similar thoughts and the counselling was mentioned to have improved the knowledge of the parents about the intervention^(12)^. A recent meta-analysis that reviewed 13 randomised controlled trials of psychosocial stimulation interventions in Low- and Middle- Income Countries also reported similar findings, where the intervention improved maternal knowledge about the development needs of children^(27)^. Similarly, health workers also reported the benefit of the interventions in terms of enhancing the outcome of hospitalised children and expressed their positive attitude.

Moreover, most parents expressed positive experiences with participating in psychosocial stimulation interventions for children with SAM, although some of them also shared their unpleasant experiences. The positive experiences of the parents were mainly attributed to the improvements in the behaviour, mood, and performance of children and enhanced interactions between the child and parents. In line with this, health and intervention workers who facilitated the intervention reported positive experiences with the implementation of the intervention. The finding highlighted the acceptability of psychosocial stimulation interventions among both parents and health workers. A study conducted in Jimma, Ethiopia reported a similar finding where psychomotor/ psychosocial stimulation was well accepted by most caregivers. The positive changes seen in children’s development and behaviour as well as improved parent-child interactions were reported as the prime factors for the acceptance of the intervention by mothers/caregivers^(10)^. Another qualitative study conducted to understand the perspectives of primary caregivers who participated in the Kusamala Program reported that the programme was viewed positively by caregivers, with many participants outlining concepts they learned and aimed to apply at home^(12)^. A pilot study conducted in rehabilitation centres across India has also reported the positive feedback of the mothers. The improvement in the children’s development after the sensory/psychosocial stimulation and structured play activities was reported as key motivator for the mothers to implement the interventions^(28)^. Health workers identified several opportunities and challenges to the effective integration of the intervention for the long-term benefits of children hospitalised with SAM. The fact that psychosocial stimulation interventions were already included in the national guidelines for SAM management, and the availability of functional SC in most public health facilities with health workers and community health workers were identified as key factors that confer the opportunity for the integration of the intervention with routine medico-nutritional care. Given the limited capacity of the health system in Ethiopia to afford the cost of resource-intensive interventions, health workers mentioned the relative cost-effectiveness of the intervention as the key contributor to the feasibility of the intervention.

Challenges such as the lack of an operational manual that guides the setting up and implementation of the intervention were reported as major challenges. Health workers mentioned that, except for little information available in the national documents, there was no document such as a guideline or manual that guides the standardisation and effective implementation of the intervention. Moreover, the lack of adequate resources, including play materials, space/play area, and other essentials needed to administer the intervention, was also mentioned as a major challenge. The shortage of health workers in most of the health facilities and the lack of capacity building training, except for the very basic elements included in the training of SAM management, were also identified. Moreover, time was also found to be the major limitation on behalf of the health workers. Lack of adequate supervision and support for service providers and lack of accountability mechanisms or incentives were also reported as challenges. Some health workers linked the lack of tools, capacity building initiatives, and accountability and supervision mechanisms toward psychosocial stimulation interventions with the inadequate priority given to the intervention at different levels of the health system.

A study conducted to assess the feasibility of implementing and sustaining the WHO guidelines for inpatient management of SAM identified that stimulation and an effective system of follow up were not implemented in under-resourced hospitals in rural South Africa. Among others, understaffing, inadequate training, high doctor turnover, nurses’ inattentiveness, and insufficient interaction with carers were reported as the constraints^(11)^. A study conducted in Malawi reported prioritisation of other activities among health workers and staff shortages as the barriers to the implementation of the Kusamala Program that integrated psychosocial stimulation with nutrition, feeding; water, sanitation, and hygiene counselling^(13)^. Another pilot study conducted in India has also reported lack of adequate resource and space in Nutrition Rehabilitation Centers for execution of the age-appropriate activities^(28)^.

Social and gendered norms such as the disproportionate household burden of women, the lack of support from husbands, and their inadequate engagement were reported to influence the effective implementation of the intervention. Despite the challenges, health workers, parents, and other participants reported the feasibility of integrating the interventions with the routine medico-nutritional care provided to hospitalised SAM children in the SC.

The study has some limitations that should be acknowledged. First, the study was conducted in one zone in Central Ethiopia, which may limit the generalizability of the findings to other settings. Second, the study used a qualitative approach that relied on self-reported data from the participants, which may introduce some biases in the data. Third, the study used a purposive sampling strategy that may not capture the diversity of the population of interest.

### Conclusion

Psychosocial stimulation intervention was found to be acceptable by parents and health workers. The intervention was also feasible for implementation in health facilities operating in resource-poor settings. However, challenges that could potentially influence the effective implementation of the interventions were identified. The findings suggest the need to allocate more resources and support for the effective implementation of psychosocial stimulation interventions with the existing care provided for children with hospitalised SAM. This may include developing and implementing guidelines, standards, and appropriate monitoring mechanisms. At the health facility level, adequate resources, including play materials, space, and other essentials, should be available to regularly implement the intervention. Training programmes should also be designed for health workers. Education and awareness programmes targeting parents and the community could also be among the relevant interventions. Finally, more studies should be conducted to understand the perspectives of key stakeholders, including policymakers, civil society organisations, donors, and others.

## References

[1] UNICEF. *Unicef Programme Guidance Document: Management of Severe Acute Malnutrition in Children: Working Towards Results at Scale*. New York, USA: UNICEF; 2015.

[2] UNICEF. *Emergency Nutrition Response in Ethiopia*. UNICEF; 2019.

[3] Ethiopian FMoH. Guidelines for the Management of Acute Malnutrition. Addis Ababa: Ethiopia; 2016.

[4] Ashworth A , Khanum S , Jackson A , et al. Guidelines for the Inpatient Treatment of Severely Malnourished Children. Geneva: World Health Organization; 2003.

[5] Levitsky D , Strupp B. The relationship between undernutrition and behavioral development in children malnutrition and the brain: changing concepts, changing concerns. J Nutr. 1995;125(8):2212S–2220S. 7542703 10.1093/jn/125.suppl_8.2212S

[6] Grantham-McGregor SM , Fernald LCH , Kagawa RMC , et al. Effects of integrated child development and nutrition interventions on child development and nutritional status. Ann N Y Acad Sci. 2013;1308:11–32. 24673166 10.1111/nyas.12284

[7] Grantham-McGregor S , Baker-Henningham H. Review of the evidence linking protein and energy to mental development. Public Health Nutr. 2005;8(7A):1191S–1201S. 10.1079/phn2005805 16277829

[8] Grantham-McGregor S , Schofield W , Powell C. Development of severely malnourished children who received psychosocial stimulation: six-year follow-up. Pediatrics. 1987;79(2):247–254. 3808797

[9] Nahar B , Hamadani JD , Ahmed T , et al. Effects of psychosocial stimulation on growth and development of severely malnourished children in a nutrition unit in Bangladesh. Eur J Clin Nutr [Internet]. 2009;63(6):725–731. 10.1038/ejcn.2008.44 (accessed June 2018). 18772893

[10] Abessa TG , Worku BN , Wondafrash M , et al. Effect of play-based family-centered psychomotor/psychosocial stimulation on the development of severely acutely malnourished children under six in a low-income setting: a randomized controlled trial. BMC Pediatr. 2019;19(1):336. 31521161 10.1186/s12887-019-1696-z PMC6744679

[11] Karaolis N , Jackson D , Ashworth A , et al. WHO guidelines for severe malnutrition: are they feasible in rural African hospitals? Arch Dis Child. 2007;92(3):198–204. 16670119 10.1136/adc.2005.087346 PMC2083437

[12] Daniel AI , Bwanali M , Tenthani JC , et al. A mixed-methods cluster-randomized controlled trial of a hospital-based psychosocial stimulation and counseling program for caregivers and children with severe acute malnutrition. Curr Dev Nutr. 2021;5(8):1–12. 34447897 10.1093/cdn/nzab100 PMC8382273

[13] Daniel AI , Van Den Heuvel M , Gladstone M , et al. A mixed methods feasibility study of the Kusamala Program at a nutritional rehabilitation unit in Malawi. Pilot Feasibility Stud. 2018;4:151. 30258650 10.1186/s40814-018-0347-8 PMC6151933

[14] Daniel AI , Bandsma RH , Lytvyn L , et al. Psychosocial stimulation interventions for children with severe acute malnutrition: a systematic review. J Glob Health. 2017;7(1):010405. 28567278 10.7189/jogh.07.010405 PMC5441448

[15] Kerac M , McGrath M , Grijalva-Eternod C , et al. Management of Acute Malnutrition in Infants (MAMI) Project: Technical Review; 2010; https://www.researchgate.net/publication/367362464_Management_of_Acute_Malnutrition_in_Infants_MAMI_Technical_Review_Current_evidence_policies_practices_programme_outcomes (accessed March 2023).

[16] CSA. Population Size by Sex, Region, Zone and Wereda. Published 2021; https://www.statsethiopia.gov.et/wp-content/uploads/2020/08/Population-of-Weredas-as-of-July-2021.pdf (accessed January 2022).

[17] Ejeta B , Ermias D , Ashetu G. Determinants of smallholder commercialization of tomato crop in Siltie Zone, Southern Ethiopia. Agric For Fish. 2020;9(6):163.

[18] EPHI, ICF. Ethiopia Mini Demographic and Health Survey 2019: Final Report. Rockville, MD: EPHI and ICF; 2021.

[19] Tessema TT , Alamdo AG , Yirtaw TG , et al. The effects of psychosocial stimulation on the development, growth, and treatment outcome of children with severe acute malnutrition age 6-59 months in southern Ethiopia: a parallel group cluster randomized control trial (EPSoSAMC study). BMC Public Health [Internet]. 2019;19(1610):1–12. 10.1186/s12889-019-7916-5 (accessed April 2021). 31791303 PMC6889618

[20] Tong A , Sainsbury P , Craig J. Consolidated criteria for reporting qualitative research (COREQ): a 32-item checklist for interviews and focus groups. Int J Qual Heal Care. 2007;19(6):349–57. 10.1093/intqhc/mzm042 17872937

[21] Braun V , Clarke V. Using thematic analysis in psychology. Qual Res Psychol [Internet]. 2006;3(2):77–101. https://psychology.ukzn.ac.za/?mdocs-file=1176 (accessed January 2023).

[22] Assefa DG , Woldesenbet TT , Molla W , et al. Assessment of knowledge, attitude and practice of mothers/caregivers on infant and young child feeding in Assosa Woreda, Assosa Zone, Benshangul Gumuz Region, Western Ethiopia: a cross-sectional study. Arch Public Heal. 2021;79(1):1–10. 10.1186/s13690-021-00690-5 PMC8466390 34563264

[23] Gebremichael B , Abate BB , Tesfaye T. Mothers had inadequate knowledge towards key essential nutrition action messages in mainly rural Northeast Ethiopia. J Nutr Sci. 2021;10:1–7. 10.1017/jns.2021.10 PMC8057394 33889402

[24] WHO, UNICEF. *Global Strategy for Infant and Young Child Feeding. 54th World Health Assembly*; 2003 May 13-18. Geneva, Switzerland: World Health Organization; 2003.

[25] Girma T , James PT , Abdissa A , et al. Nutrition status and morbidity of Ethiopian children after recovery from severe acute malnutrition: prospective matched cohort study. PLoS One [Internet]. 2022;17:1–15. 10.1371/journal.pone.0264719 (accessed May 2023). PMC8912152 35271590

[26] Dereje N. Determinants of severe acute malnutrition among under five children in Shashogo Woreda, Southern Ethiopia: a community based matched case control study. J Nutr Food Sci. 2014;4(5):300.

[27] Jeong J , Yousafzai A. Stimulation interventions and parenting in low- and middle- income countries: a meta-analysis. Pediatrics. 2018;14(4):e20173510. 10.1542/peds.2017-3510 29500293

[28] Kumar P , Rohatgi S , Singh P , et al. Strengthening psychosocial stimulation in the management of children with severe acute malnutrition: experience from a nutrition rehabilitation center. Indian Pediatr. 2021;58:42–45. 34687188

